# Supplementary material for: Reactive Metamizole Metabolites Enhance the Toxicity of Hemin on the ATP Pool in HL60 Cells by Inhibition of Glycolysis
Source: Biomedicines. 2020 Jul 14;8(7):212. doi: 10.3390/biomedicines8070212 (PMC7400389; doi:10.3390/biomedicines8070212)
Supplement: Supplementary file 1 [file biomedicines-08-00212-s001.pdf]

Supporting results

Reactive metamizole metabolites enhance the toxicity of hemin on the ATP pool in HL60 cells by inhibition of glycolysis

Deborah Rudin[a,b], Maurice Schmutz[a,b], Noëmi Johanna Roos[a,b], Jamal Bouitbir[a,b,c], Stephan Krähenbühl[a,b,c*]

deborah.rudin@unibas.ch, maurice.schmutz@unibas.ch, jamal.bouitbir@unibas.ch, stephan.kraehenbuehl@usb.ch

[a]Division of Clinical Pharmacology & Toxicology, University Hospital Basel, Switzerland

[b]Department of Biomedicine, University of Basel, Switzerland

[c]Swiss Centre for Applied Human Toxicology (SCAHT), University of Basel, Switzerland

***Corresponding author**

Stephan Krähenbühl, MD, PhD

Clinical Pharmacology & Toxicology

University Hospital

4031 Basel

Tel: +41 61 265 4715

Fax: +41 61 265 4560

e-mail: stephan.kraehenbuehl@usb.ch

Supplementary Figure S1

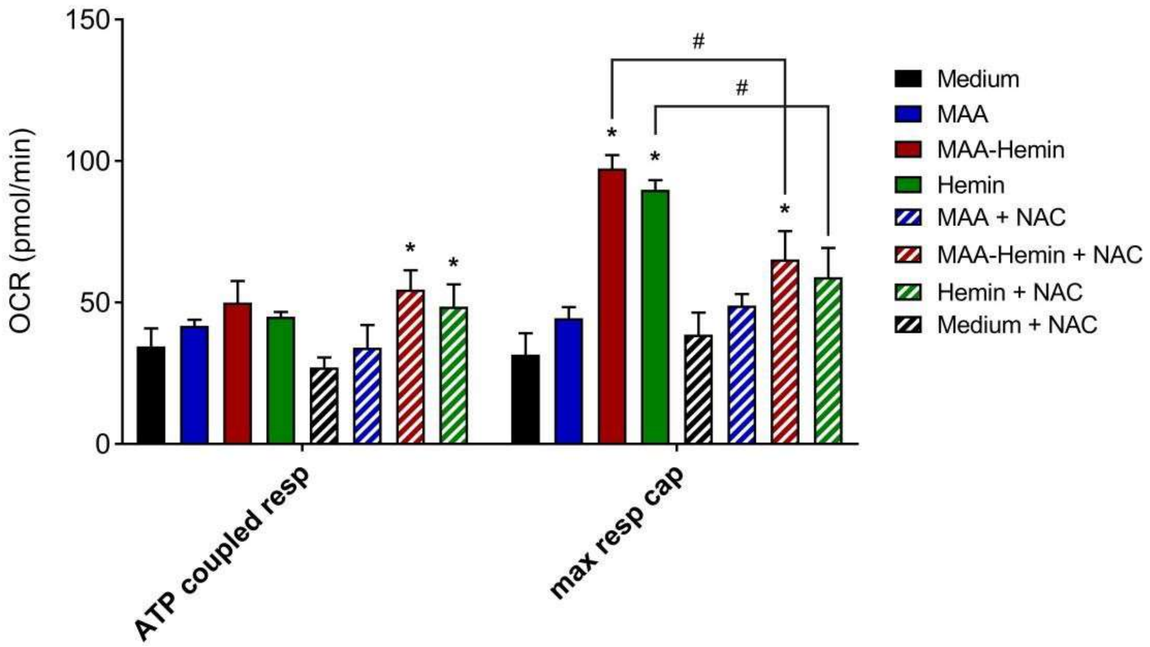

Figure S1. *Quantification of the effect of MAA and/or hemin on cellular oxygen consumption (quantification of the results shown in Figure 2A and 2B)*. HL60 cells were cultured in the presence of 10 mM glucose and treated with the toxicants for 24 hours. ATP coupled respiration was calculated as the difference between basal respiration and proton leak and maximal respiration as the respiration in the presence of FCCP minus respiration in the presence of rotenone as shown in Figure 2. Concentrations used were MAA 100 µM, hemin 12.5 µM and N-acetylcysteine (NAC) 1 mM. Results are presented as mean ± SEM of n=3 independent experiments in triplicate.

*$p<0.05$ vs. medium-treated control incubations of the same group. $^{\#}p>0.05$ vs. respective incubations containing NAC.

Supplementary Figure S2

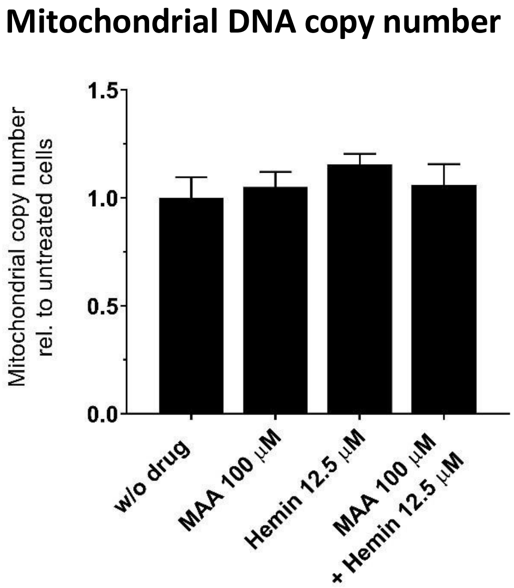

Figure S2. *Effect of MAA and/or hemin on the mitochondrial DNA copy number*. HL60 cells were cultured in the presence of 10 mM glucose and treated with the toxicants for 24 hours. The mitochondrial DNA copy number was determined as the mtDNA/nDNA ratio as described by Quiros et al. (Curr Protoc Mouse Biol 2017;7:47-54). There were no significant differences between the groups investigated.

Supplementary Figure S3

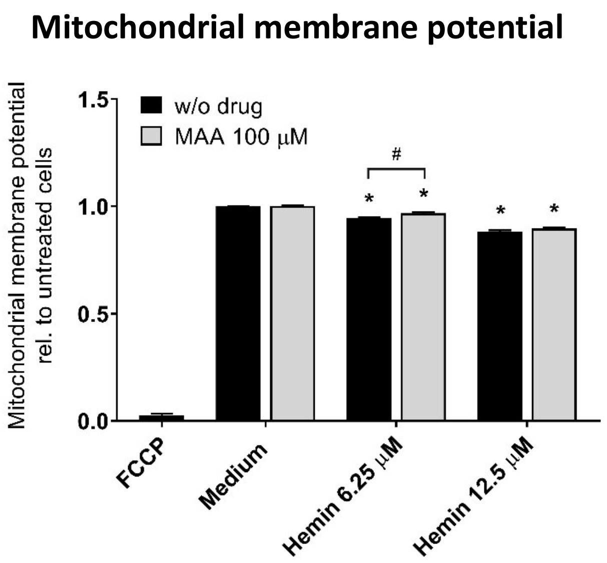

Figure S3 *Effect MAA and/or hemin on the mitochondrial membrane potential*. HL60 cells were cultured in the presence of 10 mM glucose and treated with the toxicants for 24 hours. The effect of the toxicants on the mitochondrial membrane potential assessed using tetramethylrhodamine methyl ester (TMRM). The uncoupler carbonyl cyanide 4-(trifluoromethoxy)phenylhydrazone (FCCP) was used as a positive control. Results are presented related to vehicle-treated control incubations. N=3 independent experiments in triplicate. *p<0.05 vs. vehicle-treated control incubations, #p<0.05 vs. the corresponding value of incubations without hemin.

.

Supplementary Figure S4

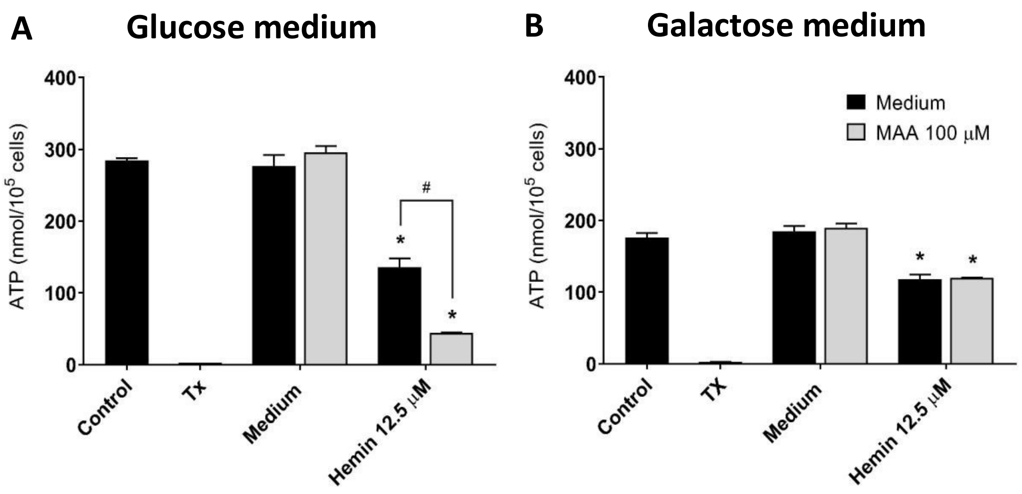

Figure S4 *Effect MAA and/or hemin on the cellular ATP content*. HL60 cells were treated with the toxicants for 24 hours. Cells were cultured in the presence of 10 mM glucose or in the presence of 10 mM galactose in order to force the cells to mitochondrial ATP generation. **A**. Cellular ATP content determined using the CellTiter-Glo® luminescent assay in cells cultured in 10 mM glucose. **B**. Cellular ATP content determined using the CellTiter-Glo® luminescent assay in cells cultured in 10 mM galactose. *p<0.05 vs. vehicle-treated control incubations, #p<0.05 vs. the corresponding value of incubations without hemin.
